# Discriminative application of string similarity methods to chemical and non-chemical names for biomedical abbreviation clustering

**DOI:** 10.1186/1471-2164-13-S3-S8

**Published:** 2012-06-11

**Authors:** Atsuko Yamaguchi, Yasunori Yamamoto, Jin-Dong Kim, Toshihisa Takagi, Akinori Yonezawa

**Affiliations:** 1 Database Center for Life Science, Bunkyo-ku, Tokyo, Japan; 2 Department of Computational Biology, The University of Tokyo, Kashiwa, Chiba, Japan

## Abstract

**Background:**

Term clustering, by measuring the string similarities between terms, is known within the natural language processing community to be an effective method for improving the quality of texts and dictionaries. However, we have observed that chemical names are difficult to cluster using string similarity measures. In order to clearly demonstrate this difficulty, we compared the string similarities determined using the edit distance, the Monge-Elkan score, SoftTFIDF, and the bigram Dice coefficient for chemical names with those for non-chemical names.

**Results:**

Our experimental results revealed the following: (1) The edit distance had the best performance in the matching of full forms, whereas Cohen et al. reported that SoftTFIDF with the Jaro-Winkler distance would yield the best measure for matching pairs of terms for their experiments. (2) For each of the string similarity measures above, the best threshold for term matching differs for chemical names and for non-chemical names; the difference is especially large for the edit distance. (3) Although the matching results obtained for chemical names using the edit distance, Monge-Elkan scores, or the bigram Dice coefficients are better than the result obtained for non-chemical names, the results were contrary when using SoftTFIDF. (4) A suitable weight for chemical names varies substantially from one for non-chemical names. In particular, a weight vector that has been optimized for non-chemical names is not suitable for chemical names. (5) The matching results using the edit distances improve further by dividing a set of full forms into two subsets, according to whether a full form is a chemical name or not. These results show that our hypothesis is acceptable, and that we can significantly improve the performance of abbreviation-full form clustering by computing chemical names and non-chemical names separately.

**Conclusions:**

In conclusion, the discriminative application of string similarity methods to chemical and non-chemical names may be a simple yet effective way to improve the performance of term clustering.

## Background

Clustering terms based on string similarity is a common task in text processing and is used to abstract varying of representations of the same concept in natural language texts. To address the task, several string similarity methods have been developed and have been successfully applied [1].

When we apply similarity methods, at least two problems arise: (1) the choice of a good similarity method, and (2) the choice of an optimal threshold. For example, Cohen et al. [2] reported that SoftTFIDF generally works the best for the term clustering of entity names, and Okazaki et al. [3] reported that the use of a hybrid distance with 0.2 as the optimal threshold was the best setup for the problem of abbreviation-full form clustering.

The work presented in this paper was carried out as a part of a dictionary-building project for abbreviations in life science. The project was motivated by the observation that abbreviated terms are abundant in life science literature and that there is a significant need for a dictionary lookup service for such abbreviated terms.

It has been reported that a new abbreviation appears in every five to ten abstracts in PubMed [4], and [5] showed that the number of MEDLINE entries increased by approximately 650 000 entries per year on average from 2004 to 2009. These facts indicate the necessity for an abbreviation dictionary to be continuously updated, thus implicating the necessity for an automated process to extract abbreviations from texts in MEDLINE and integrate them into the existing dictionary entries. There have been several studies in which such systems were developed [4,6-9]. These systems typically employ two processes: (1) the extraction of abbreviation-full form terms, and (2) the clustering of these terms per their meanings. Our focus in this paper is the clustering problem.

We have been developing and maintaining the Allie database, in addition to an online service that provides abbreviation-full form information, by referencing PubMed entries and the subject domains in which they appear. Allie is updated monthly to include new abbreviated terms that are found in PubMed. Because new abbreviations are constantly added to the database, the clustering of abbreviation-full forms also needs to be updated. Therefore, we have been developing an automatic term-clustering method. There have been several works sharing this same goal [3,10,11].

We have tested several similarity methods. We observed a significant difference in the distribution of string similarities between terms according to the semantic classes of those terms. In particular, we focused on chemical names that seldom allow even small variations in spelling to qualify as a matching. For example, although both *diethylene glycol monoethyl ether *and *diethylene glycol monomethyl ether *are abbreviated as *DGME *in MEDLINE abstracts and the difference between these terms is only the insertion of a single character, *m*, these terms denote different chemical compounds. The motivation of our study described in this paper was to solve this problem.

In this study, we proposed the following hypothesis: "chemical names and other terms have different distributions of character sequences; thus, the computation of their similarities should be carried out in different ways."

To argue this hypothesis, in this study, we compared the results of four string measures for chemical names with the results for the other full forms. The four measures used were the edit distance, the Monge-Elkan score, SoftTFIDF with the Jaro-Winkler distance, and the bigram Dice coefficient.

## Methods

### Similarity measures

For the clustering of full forms that share the same abbreviation, we chose to test four similarity measures: the length-normalized edit distance, the Monge-Elkan score, SoftTFIDF with the Jaro-Winkler distance, and the Dice coefficient based on character bigrams. The selection of these measures was motivated by their popularity (edit distance), performance reported in [2] (Monge-Elkan and SoftTFIDF) and simplicity (Dice coefficient).

The edit distance, also known as the Levenshtein distance, is one of the most commonly studied string distance measures. The edit distance of two strings is defined as the minimum number of edit operations to transform one string into the other string, where an edit operation is an insertion, a deletion, or the replacement of a single character. In this study, we employed the length-normalized edit distance, defined as follows, to eliminate the influence of the length of the full forms:

$$d\left(s_{1},s_{2}\right)=ed\left(s_{1},s_{2}\right)/)\max\left\{n_{1},n_{2}\right\}$$

where *ed*(*s*_1_, *s*_2_) indicates the edit distance between two strings *s*_1 _and *s*_2_, and *n*_1 _and *n*_2 _are the lengths of *s*_1 _and *s*_2_. Because the Levenshtein distance between *s*_1 _and *s*_2 _is computable by dynamic programming with *O*(*n*_1_*n*_2_) [12], the length-normalized edit distance is computable with the same order.

The Monge-Elkan score [13] is another alignment-based similarity measure. This measure is defined as the minimum sum of the scores for all possible alignments of two strings. A score matrix for the Monge-Elkan is {5, 2, -5}, where the result is 5 if two characters are the same, 2 if two characters are in one set of {*d*, *t*}, {*g*, *j*}, {*l*, *r*}, {*m*, *n*}, {*b*, *p*, *v*}, or {*a*, *e*, *i*, *o*, *u*}, and -5, otherwise. In addition, an affine gap penalty is defined as *g*(*k*) = *α *+ *βk*, with *α *= 5 and *β *= 1. Note that if you employ the score matrix {0, -1, -1} and the gap penalty *g*(*k*) = *k*, the score is equal to -*d *where *d *is the edit distance. In [2], the Monge-Elkan score was reported to perform the best among alignment-based measures in most cases, if the score matrix {5, 3, -3} is used, and if the score is scaled to the interval [0, 1]. Therefore, in our experiment, we also employed this score matrix as the Monge-Elkan score.

The SoftTFIDF, which was introduced by [2], is a variation of TFIDF, but allows approximate string matchings of words, instead of only allowing exact matchings. The SoftTFIDF with a similarity measure *s*' for a certain set *S *of strings is defined by:

$$s\left(s_{1},s_{2}\right)=\underset{w\in sw\left(s_{1},s_{2}\right)}{\sum }Vs\left(w,s_{1}\right)\times Vs\left(w,s_{2}\right)\times \underset{w_{i}\in s_{2}}{\max}\;s^{\prime }\left(w,w_{i}\right)$$

where *sw*(*s*_1_, *s*_2_) is set of words in string *s*_1 _such that, for each word *w *in *sw*(*s*_1_, *s*_2_), there exists a word *w*' in string *s*_2 _such that *s*'(*w*, *w*') is at least a given constant *a*,

$$Vs\left(w,s\right)=\frac{\log\left(TF\left(w,s\right)+1\right)\times \log\left(IDFs\left(w\right)\right)}{\sqrt{\sum_{w_{i}}{\left(\log\left(TF\left(w,s\right)+1\right)\times \log\left(IDFs\left(w\right)\right)\right)}^{2}}}$$

where *T F *(*w*, *s*) is the frequency of the word *w *in *s*, and *IDF_S_*(*w*) is the inverse of the fraction of strings in *S *that contain the word *w*. Because they employed the Jaro-Winkler score as the similarity measure *s*' and 0.9 as the constant *a *in their experiment, we also employed these values in our experiment.

The Jaro score is defined as follows:

$$s_{Jaro}\left(s_{1},s_{2}\right)=\frac{1}{3}\left(\frac{n_{1}^{\prime }}{n_{1}}+\frac{n_{2}^{\prime }}{n_{2}}+\frac{n_{1}^{\prime }-T_{s_{1},s_{2}}}{2n_{1}^{\prime }}\right)$$

where $n_{1}^{\prime }$ and $n_{2}^{\prime }$ are the numbers of matching characters in *s*_1 _and *s*_2_, respectively, where a character in one string is matching if the same character is present in the other string, and they are not farther than min(*n*_1_, *n*_2_)/2 apart. Then, the Jaro-Winkler score is

$$s_{JW}\left(s_{1},s_{2}\right)=s_{Jaro}\left(s_{1},s_{2}\right)+\frac{max\left\{p,4\right\}}{10}\left(1-s_{Jaro}\left(s_{1},s_{2}\right)\right),$$

where p is the number of common prefix characters between *s*_1 _and *s*_2_.

N-gram analysis is also frequently used as a string similarity measure for various purposes [14-17]. Bigrams or trigrams are mainly used as a string similarity measure for clustering terms. In our initial experiment, we found that bigrams are better than trigrams, for our purposes. Therefore, we employed bigrams in our experiment. The similarity used in this paper is the Dice coefficient, defined as follows [14]:

$$s_{n}\left(s_{1},s_{2}\right)=2\times c_{n}\left(s_{1},s_{2}\right)/)\left(n_{1}+n_{2}\right)$$

where *c_n_*(*s*_1_, *s*_2_) indicates the number of substrings of length *n *in *s*_1 _that match length *n *substrings in *s*_2_. Note that the edit distance is a distance measure, whereas the others are similarity measures. Thus, the lower the edit distance, and the higher the other similarities, the better the chance that the two strings will be clustered.

### Term clustering

The problem we want to address is the clustering of the full form terms corresponding to abbreviations based on their string similarities.

We assume that every term *s *is assigned to be a hidden element in a certain set of concepts. Many methods for clustering terms are based on predicating whether two terms are mapped to the same concept or not. Therefore, we cast the problem as a binary decision task, to determine whether to cluster two given terms. This decision was made based on a similarity measure and a threshold as a cutoff point. A hybrid model combining multiple similarity measures was not considered, since the purpose of this work was to test the effect of different similarity measures when applied to different groups of terms.

With the task setting, our goal was, for a given set of terms, to identify the similarity measure and the threshold value that yielded the best set of matchings between two terms (i.e., the set that best agreed with the set that was obtained by matching two terms that were mapped to the same concepts).

### Data preparation

This section describes the data-set that we prepared for the abbreviation-full form clustering experiment. We defined the pair consisting of an abbreviation and its full form, as an A-pair. We considered two A-pairs to be mapped to each other when (1) they shared the same abbreviation, and (2) the full forms belonged to the same concept class. The goal of our experiment was, for pairs of A-pairs with the same abbreviation, to compare the performances of the clustering methods using a string similarity of the full forms between chemical names and non-chemical names.

**Figure 1:**
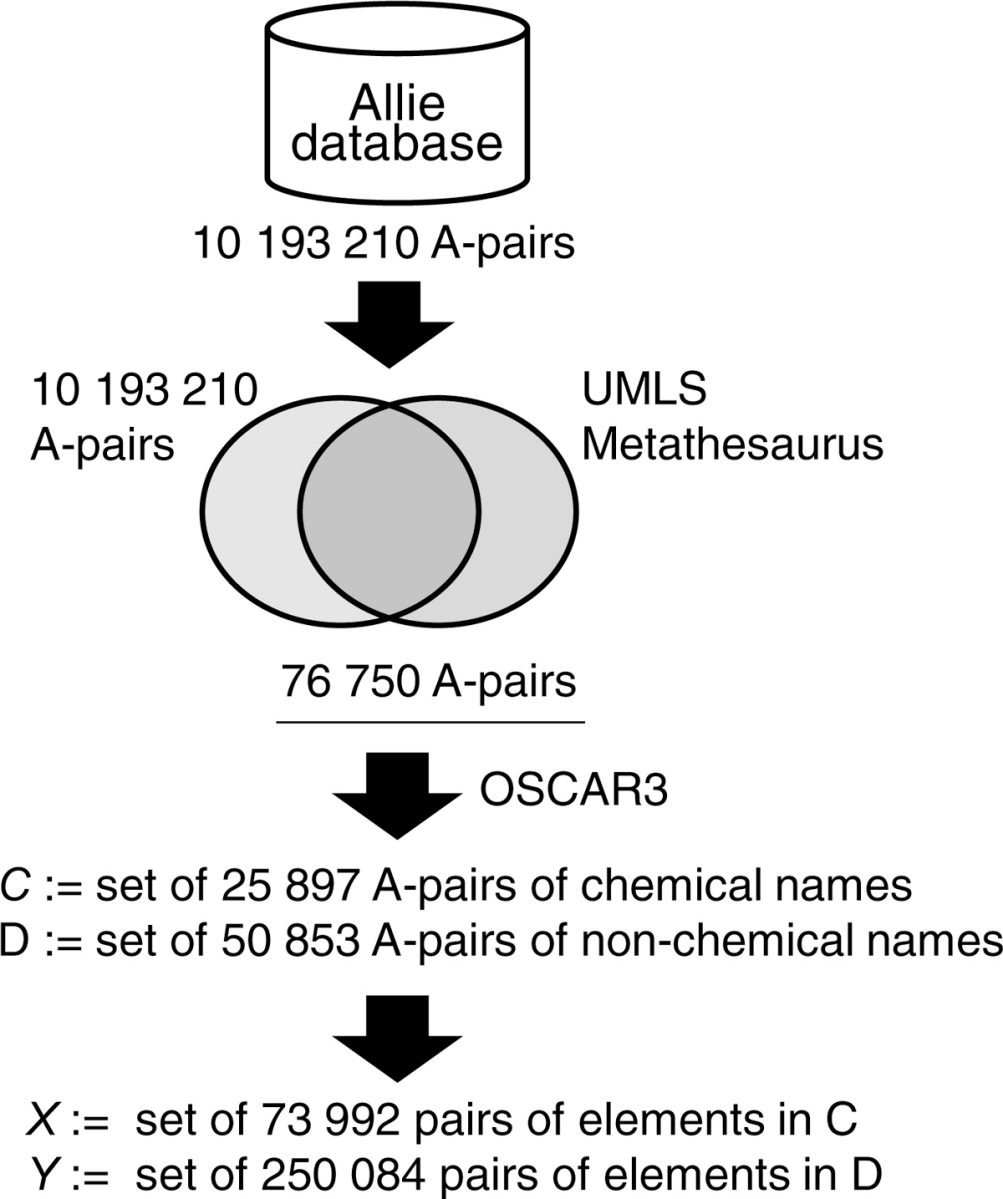
**Dataset preparation**. The flowchart of the process used to obtain datasets *X *and *Y *for our experiment.

Figure 1 illustrates the process by which we prepared the data sets for experiments. The goal was to prepare two sets of A-pairs, one for chemical names (set C), and the other for non-chemical names (set D). To evaluate the performance of automatic clustering, we needed a gold standard for clustering.

We began with the set of A-pairs (10 193 210 entries) obtained from the current Allie database. Among the entries, we collected the A-pairs for which the full form appears in the UMLS Metathesaurus [18] with CUI (Concept Unique Identifier). The UMLS Metathesaurus is the largest thesaurus in the biological domain, and includes 2 404 937 concepts in the current version (2011AA). The CUI was then used to determine the fold clustering of the collected A-pairs (76 750 entries). Because we wanted to compare the performances of the similarity measures for chemical and non-chemical names, we divided the set of A-pairs with the gold standard of clustering into two subsets: one containing chemical names (set C) and the other containing non-chemical names (set D). To identify chemical names, we used OSCAR3 [19]. In a set of A-pairs, all A-pairs sharing the same abbreviation were candidates for mapping. We found 73 992 and 250 084 pairs (of A-pairs) in the C and D sets, respectively.

In our preliminary experiment, we confirmed that the frequencies of each letter for chemical names and non-chemical names were similar. Therefore, the results should be minimally impacted by the difference of the letter frequency distributions between chemical names and non-chemical names.

### Experimental setup

We experimented with the two sets *X *and *Y *of mapping candidates. For each pair of mapping candidates (i.e., a pair of A-pairs sharing the same abbreviation), the gold mapping, true or false, was obtained using the CUI. If the CUIs of the full forms of both A-pairs were the same, then the mapping was true; otherwise, the mapping was false. In a series of experiments, similarity measures were used to predict the mapping, and the performance was evaluated by comparing the predictions with the gold mappings. We first computed the four string measures described in the Subsection "Similarity measures" for all the pairs, in both *X *and *Y*. After that, for each string measure, we computed the recalls, precisions, and F-measures of the matchings of chemical names for every 0.05 threshold from 0.0 to 1.0 or from 1.0 to 0.0. Similarly, we computed those values for the non-chemical names. In addition, for SoftTFIDF, we computed these values for every 0.005 threshold from 1.0 to 0.9, since the peak F-measure for SoftTFIDF was unclear when using the 0.05 threshold.

Furthermore, we constructed two 26-dimensional vectors, each element of which indicates a weight of an edit operation of an insertion or a deletion of a character from 'a' to 'z' for the length-normalized edit distance. One vector is optimized by chemical names, and the other is optimized by non-chemical names. We compared the F-measures of the matchings computed by using these two weight vectors for chemical names and non-chemical names.

Finally, we compared the performances of the two methods. In the first method, all full forms were matched using the edit distance with the same threshold. In the second method, after dividing the set of full forms into two subsets according to whether a full form is a chemical name or not, the full forms were matched using different thresholds for the two subsets.

## Results and discussion

**Figure 2:**
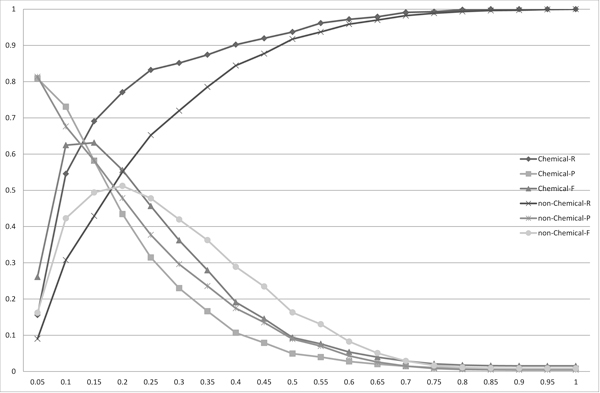
**The distribution of the recalls (R), precisions (P) and F-measures (F) for the matchings of the chemical names (Chemical) and the non-chemical names (non-Chemical) obtained using the edit distance**. The *x*-axis corresponds to the threshold used to obtain matchings.

Figure 2 shows the precision, recall, and F-measure of the mapping performance using the normalized edit distance for every 0.05 step in the threshold. The best F-measure performance was found at the thresholds of 0.125 and 0.21428 in the experimental sets *X *and *Y *, respectively. These results suggest that it is more favorable to accept more spelling variations with non-chemical names to find a good mapping than with chemical names; further, the optimal threshold was more flexible with non-chemical names, whereas the performance quickly dropped around the optimal threshold with chemical names. Therefore, we must be more strict in choosing the threshold for chemical names.

**Figure 3:**
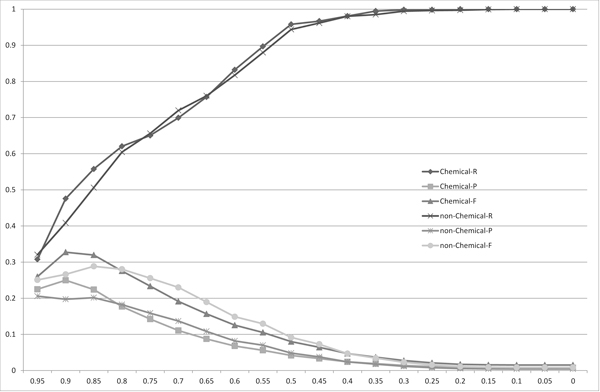
**The distribution of the recalls (R), precisions (P) and F-measures (F) for the matchings of the chemical names (Chemical) or the non-chemical names (non-Chemical) obtained using the Monge-Elkan score**. The *x*-axis corresponds to the threshold used to obtain matchings.

**Figure 4:**
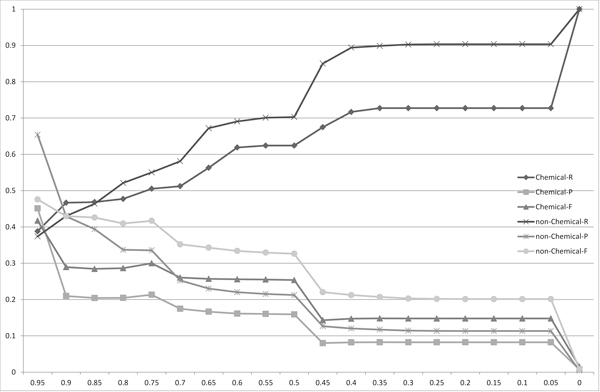
**The distribution of the recalls (R), precisions (P) and F-measures (F) for the matchings of the chemical names (Chemical) or the non-chemical names (non-Chemical) obtained using SoftTFIDF**. The *x*-axis corresponds to the threshold used to obtain matchings.

**Figure 5:**
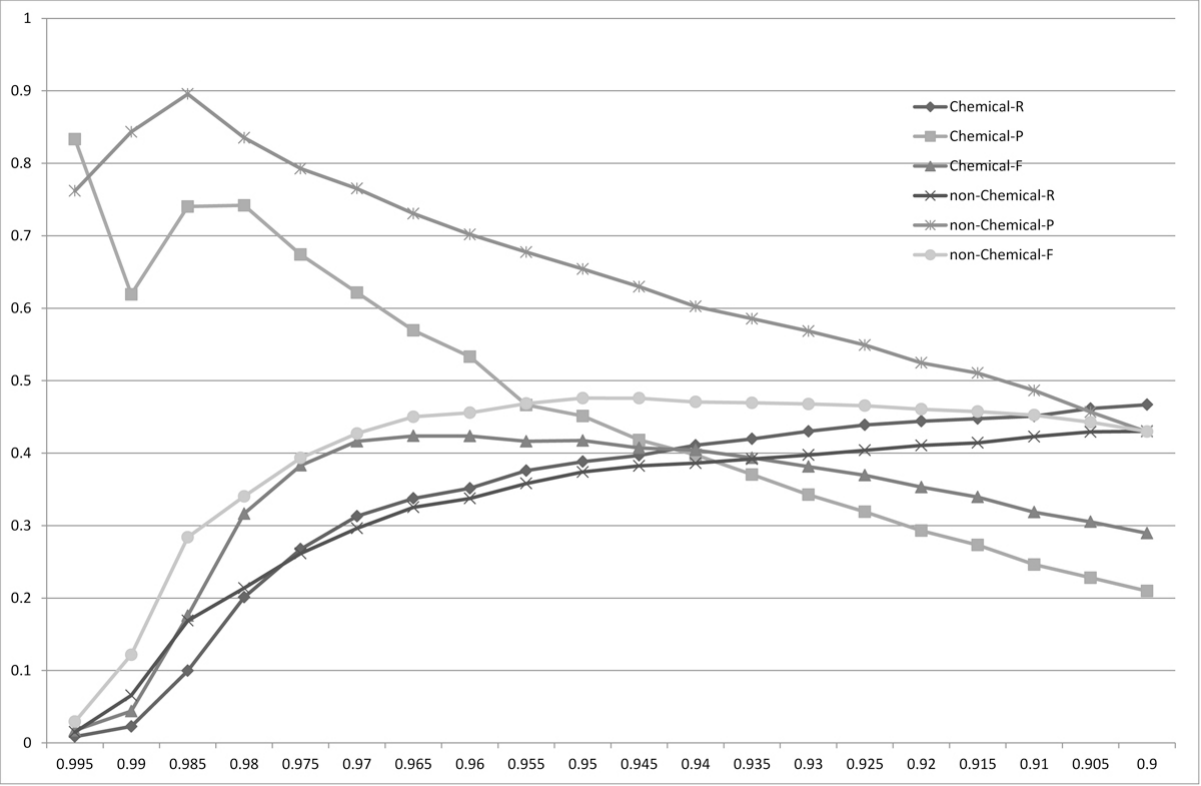
**The distribution of the recalls (R), precisions (P) and F-measures (F) for the matchings of the chemical names (Chemical) or the non-chemical names (non-Chemical) obtained using SoftTFIDF with the threshold scale of 0.005 from 0.9 to 0.995**. The *x*-axis corresponds to the threshold used to obtain matchings.

**Figure 6:**
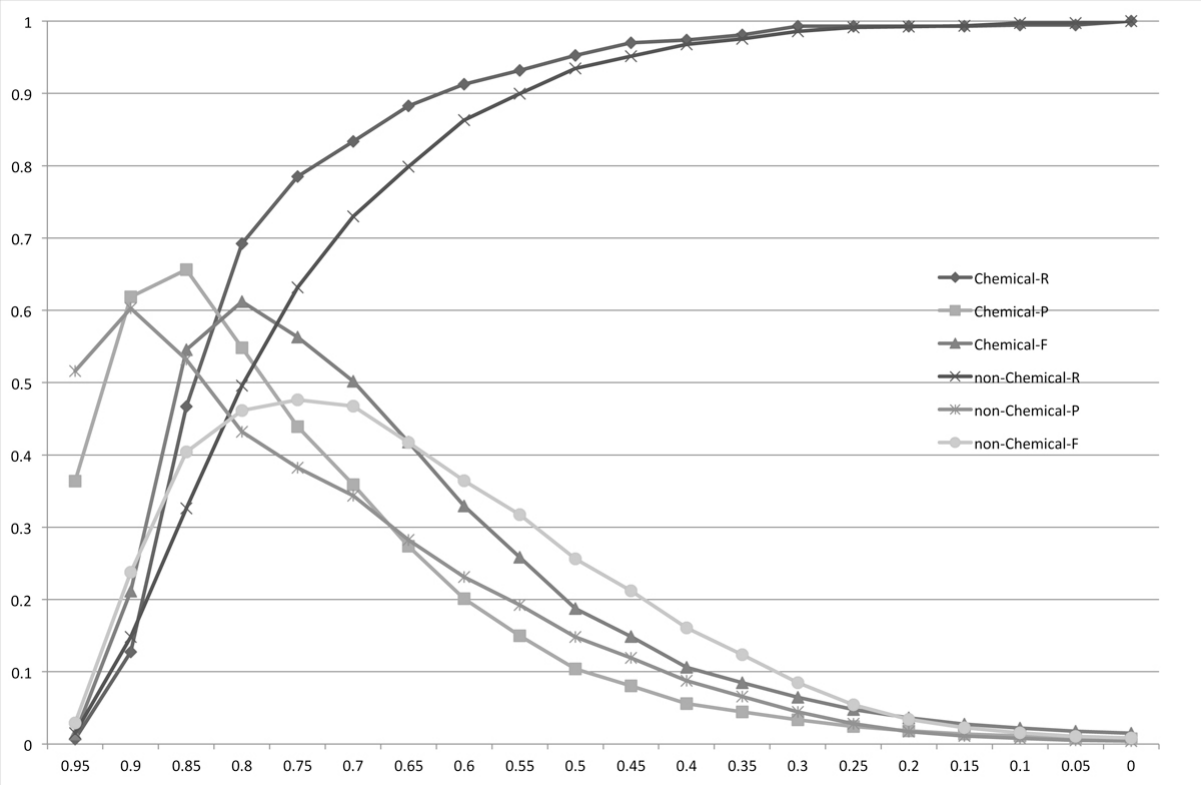
**The distribution of the recalls (R), precisions (P) and F-measures (F) for the matchings of the chemical names (Chemical) or the non-chemical names (non-Chemical) obtained using the bigram Dice coefficient**. The *x*-axis corresponds to the threshold used to obtain matchings.

Figure 3, 4, 5 and 6 show the experimental results using the Monge-Elkan score, SoftTFIDF (two figures: one is the chart plotted from 0.1 to 0.9 and the other is from 0.9 to 0.995), and the bigram Dice coefficient. Although the results from these similarity measures are less explicit, they agree with the tendency observed with the length-normalized edit distance. It is notable that SoftTFIDF generally worked better for non-chemical name terms, whereas the other similarity measures worked better for chemical names. Thus, this result suggests that SoftTFIDF may be suitable for flexible matching.

Table 1 shows the thresholds, precisions, recalls and F-measures when the F-measures are maximized to compare the recalls, precisions and F-measures among the four string similarity measures. The length-normalized edit distance had the highest F-measure among the four measures, for the both candidate sets *X *and *Y*. This result is contrary to results reported in [2], which states: the Monge-Elkan score is the best among alignment-based measures, the Levenshtein distance is considerably worse than the Monge-Elkan score, and SoftTFIDF is the best overall distance measure for their dataset. However, based on the results presented in Figures 2, 3, 4, 5, 6 and Table 1 we can see that the performances of the method using the different measures differed greatly between their dataset and ours.

**Table 1:** Comparison of the precisions, recalls and F-measures among the four methods when the F-measures were maximized

Method	Data	Precision	Recall	F-measure	Threshold
Edit	*X*	0.66857	0.61363	0.63992	*<*0.125
Distance	*Y*	0.46385	0.57731	0.51440	*<*0.21428

Monge-	*X*	0.25196	0.50524	0.33624	*>*0.88571
Elkan	*Y*	0.19872	0.58388	0.29652	*>*0.8125

Soft	*X*	0.536	0.35139	0.42449	*>*0.96047
TFIDF	*Y*	0.66222	0.37300	0.47721	*>*0.95113

Bigram	*X*	0.56086	0.67657	0.61331	*>*0.8
Dice	*Y*	0.37227	0.67197	0.47911	*>*0.73170

**Figure 7:**
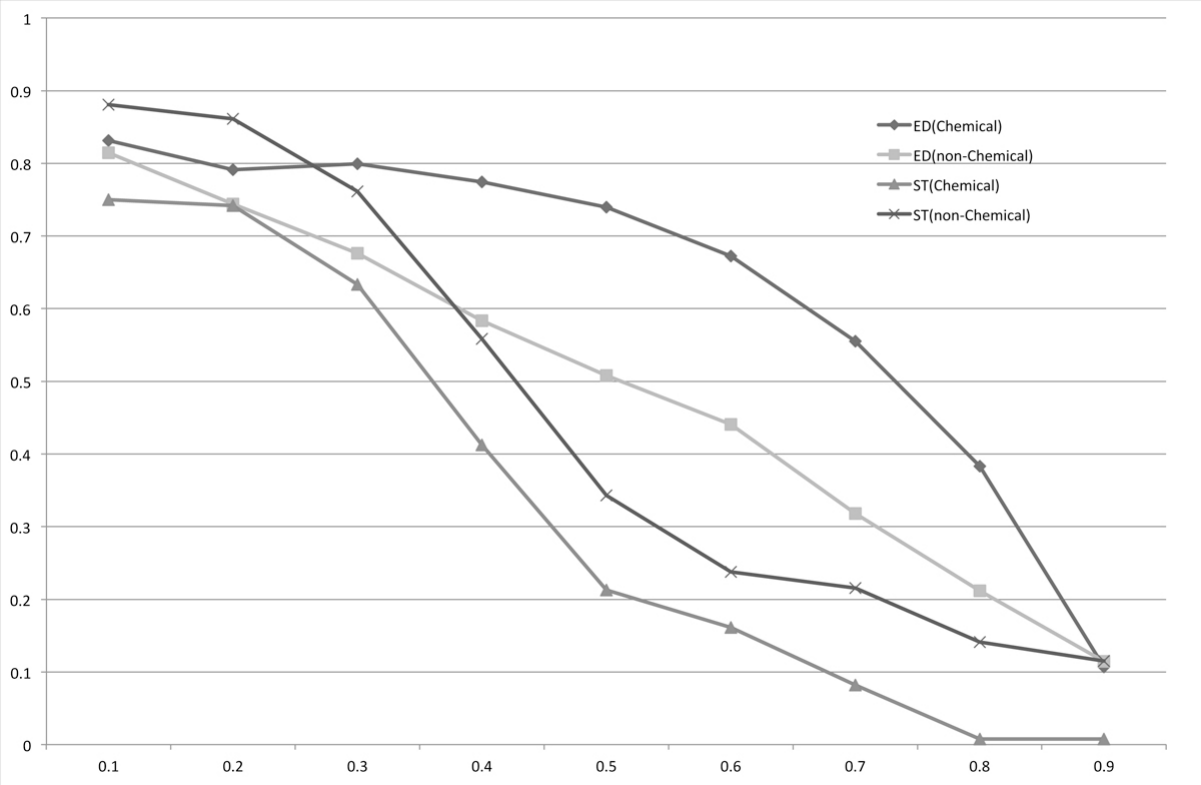
**PR curves for the length-normalized edit distance (ED) and SoftTFIDF (ST)**. We plotted recalls on the *x*-axis and precision on the *y*-axis. Chemical and non-Chemical correspond to the two datasets, the chemical names and the non-chemical names, respectively.

**Figure 8:**
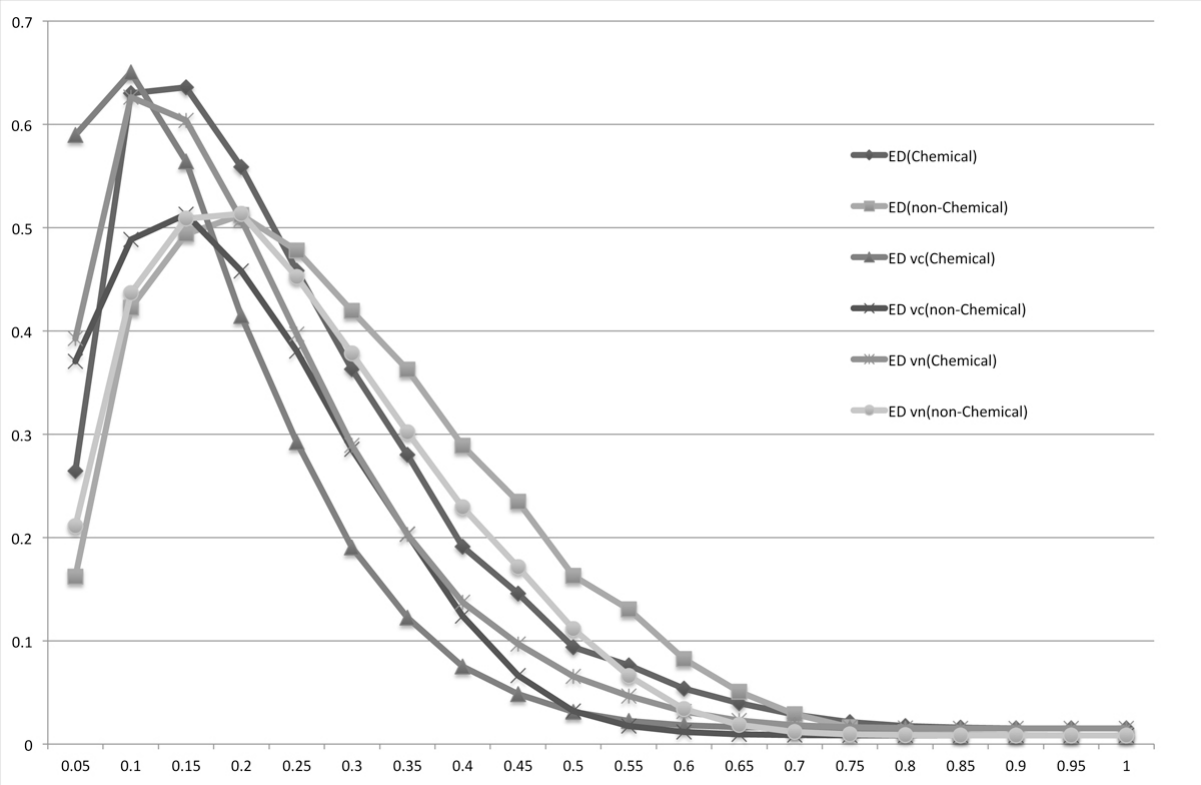
**F-measures for the matchings of the chemical names (Chemical) or the non-chemical names (non-Chemical) obtained using the length-normalized edit distance (ED), weighted ED using *v_c _*(ED vc), and weighted ED using *v_n _*(ED vn)**.

We compared the length-normalized edit distance with the SoftTFIDF result by plotting PR curves (Figure 7). As shown in this chart, SoftTFIDF is unsuitable for use with chemical names, whereas the length-normalized edit distance is suitable for chemical names. For non-chemical names, the difference between the two methods was smaller: although the maximum F-measure of the length-normalized edit distance was larger than that of SoftTFIDF, SoftTFIDF may be better if we prioritize precision. As we wrote in the Subsection "Similarity measures", the essential difference between the edit distance and the Monge-Elkan score is the weight of the score for an operation. Because we could obtain the best F-measure for both *X *and *Y *datasets by applying the length-normalized edit distance, we considered the weighted version of the length-normalized edit distance. To simplify our analysis, in this paper, we only consider 26-dimensional weight vector whose *i*-th element corresponds to weight for an operation of an insertion or a deletion of the *i*-th character among the letters 'a' to 'z'. To show the difference of weights for computing scores between chemical names and others, we computed the two 26-dimensional weight vectors *v_c _*and *v_n_*. To compute *v_c_*, we started an initial weight vector for which all the elements are 1*:*0. Then, we selected one character, in alphabetical order. We fixed values of all the elements of the vector, with the exception of the element corresponding to the selected character, and searched the value of the element for the selected character with the highest F-measure for *X*, by changing the value of the element in 0.1 at a time. If all the characters were selected, and all the values with the highest F-measures were found, we set the vector *v_c_*. In a similar way, we computed *v_n _*for non-chemical names. Table 2 shows the two vectors: *v_c _*and *v_n_*. For the bold characters 'e', 'h', 'p', 'x', 'y', and 'z', the weight values are very different. Figure 8 and Table 3 show that the weight vector *v_c _*improved the F-measure for chemical names, and the weight vector *v_n _*improved the F-measure for non-chemical names, although *v_c _*and *v_n _*are used only for insertions and deletions. However, in comparing the three F-measures for chemical names obtained by using the non-weighted edit distance, the edit distance weighted by *v_c_*, and the edit distance weighted by *v_n_*, the F-measure obtained by *v_n _*is the lowest. It is slightly lower even than the F-measure obtained by the non-weighted version. Therefore, we can see that suitable weights are also different between chemical names and non-chemical names.

**Table 2:** Optimized weight vectors for chemical names and the others

character	a	b	c	d	e	f	g	h	i	j	k	l	m
*v_c_*	1.0	1.0	1.0	1.0	0.4	1.0	1.0	0.1	0.8	1.0	1.0	0.6	0.6
*v_n_*	1.0	0.7	0.7	0.8	1.0	1.0	1.0	0.8	0.8	1.0	1.0	0.8	1.0

character	n	o	**p**	q	r	s	t	u	v	w	**x**	**y**	**z**

*v_c_*	0.7	1.0	0.1	1.0	0.9	0.6	1.0	0.2	1.0	1.0	1.0	0.3	1.0
*v_n_*	0.7	1.0	1.0	1.0	1.0	1.0	1.0	0.4	0.8	1.0	0.0	0.8	0.0

The vector *v_c _*indicates the optimized weight vector for chemical names when operations of insertions and deletions of edit distance are weighted from 0.0 to 1.0. Similarly, the vector *v_n _*indicates the optimized weight vector for non-chemical names.

**Table 3:** Comparison of the precisions, recalls and F-measures among the length-normalized edit distance, weighted edit distance using ***v_c_***, and weighted edit distance using ***v_n_***, when the F-measures were maximized

Method	Data	Precision	Recall	F-measure	Threshold
Edit	*X*	0.66857	0.61363	0.63992	*<*0.125
Distance	*Y*	0.46385	0.57731	0.51440	*<*0.21428

Weighted	*X*	0.69673	0.63461	0.66422	*<*0.08571
ED (*v_c_*)	*Y*	0.51077	0.53327	0.52177	*<*0.14117

Weighted	*X*	0.61412	0.65384	0.63336	*<*0.125
ED (*v_c_*)	*Y*	0.46225	0.60262	0.52318	*<*0.19473

Finally, to support our hypothesis presented in the Section "Background", we compared the following two results: one result was obtained by using the length-normalized edit distance with the best threshold for *X *and *Y *combined, and the other result was obtained using the best threshold for *X *and the best threshold for *Y*. To simplify the comparison, we fixed the recall at 0.8. Then, we were able to compute the threshold for *X *by sorting elements in *X *by the length-normalized edit distance, and for each *i*(0 ≤ *i *≤ |*X*|), by computing the recall when the top *i *elements are selected as matched. Table 4 provides the thresholds and precisions when recalls were the closest to 0.8: the results indicate that we can obtain a better result by simply dividing chemical names and non-chemical names into separate sets.

**Table 4:** Comparison of the precisions with a fixed recall (0.8) for the length-normalized edit distance

	Precision	Threshold
Chemical name	0.383	*<*0.222
The others	0.211	*<*0.368
All	0.25	-
Mixed	0.207	*<*0.333

## Conclusions

String similarity measures are frequently used to absorb the surface variation of terms; e.g., spelling variations, inflections, and derivations. A typical assumption is that the terms belong to the same language, and that the distribution of the characters is fixed. However, the distributions of characters used in chemical names and those used in non-chemical names vary significantly, because chemical names are often generated based on particular nomenclature systems, such as IUPAC. Based on this observation, we proposed a hypothesis: "chemical names and other terms have different distributions of character sequences; thus, the computation of their similarities should be carried out in different ways." To test the hypothesis, we conducted a series of experiments that can explicate the difference. The results strongly support this hypothesis.

We performed experimental comparisons of chemical names and other full forms based on the length-normalized edit distance, the Monge-Elkan score, SoftTFIDF and the bigram Dice coefficient. We demonstrated that (1) the length-normalized edit distance method performs the best when matching full forms according to our data; (2) for any string similarity measure above, the optimal thresholds by which to group terms differ between chemical and non-chemical names; (3) the matching method using SoftTFIDF performed better for non-chemical names than for chemical names, whereas the opposite results were obtained for the other three measures; (4) the weight vectors optimized by using non-chemical names is not suitable for chemical names; and (5) the matching result using the edit distances further improved by dividing a set of full forms into two subsets according to whether a full form is a chemical name or not. These results indicate that the distributions of the string similarities of semantically similar terms are different between chemical names and non-chemical names; thus, methods using string similarities can be potentially improved by dividing a set of terms into two sets: one consisting of chemical names and the other consisting of non-chemical names, and applying different similarity measures and different thresholds for these two sets.

It would be benefical to expand the domains of full forms including: gene names, protein names, disease names, etc. To do so, some non-trivial tasks must be completed. Such tasks include: determining how to divide appropriate domains and determining the appropriate way to divide terms into the domains. To define term domains, information such as the top 16 categories ("Anatomy", "Organisms", "Disease, Chemicals and Drugs", and so on) of MeSH (Medical Subject Headings) may be helpful. In addition, providing suitable string similarity measures, along with providing parameters for each domain, remains as a task to be completed in the future.

From an engineering perspective, a hybrid model incorporating multiple similarity measures in combination, e.g. support vector machines (SVMs), is more popular than using individual models. Our plan is to implement a hybrid model. The hypothesis confirmed in this work will provide a guideline for designing an effective hybrid model.

## Competing interests

The authors declare that there are no competing interests.

## Authors' contributions

AtY designed this study, implemented the codes, and wrote the manuscript. YY provided the Allie dataset and contributed to the discussion. AtY and JK designed the experiments. TT and AkY supervised the project. All authors have approved the final manuscript.
